# Shaping new sounds

**DOI:** 10.7554/eLife.55749

**Published:** 2020-02-12

**Authors:** Timothy D Griffiths, Kai Alter, Barbara Shinn-Cunningham

**Affiliations:** 1 Biosciences InstituteNewcastle UniversityNewcastle upon TyneUnited Kingdom; 2 Neuroscience InstituteCarnegie Mellon UniversityPittsburghUnited States

**Keywords:** tuvan throat singing, acoustic phonetics, speech biomechanics, biphonation, Human

## Abstract

MRI experiments have revealed how throat singers from Tuva produce their characteristic sound.

**Related research article** Bergevin C, Narayan C, Williams J, Mhatre N, Steeves J, Bernstein JGW, Story B. 2020. Overtone focusing in biphonic Tuvan throat singing. *eLife* **9**:e50476. doi: 10.7554/eLife.50476

Many people in Tuva – a republic in southern Siberia – have the remarkable ability to sing in two different pitches at the same time (as can be seen and heard in this video of the Alash Ensemble). This form of singing, known as throat or overtone singing, was little known outside Tuva until the author Ralph Leighton wrote a book called *Tuva or Bust!* (Leighton, 1991). The book described how Leighton and his friend Richard Feynman (the Nobel prize-winning physicist) tried and failed to travel to Tuva to study throat singing and Tuvan culture. Now, in eLife, Christopher Bergevin of York University in Canada, Brad Story of the University of Arizona and co-workers report how they have used MRI to uncover how throat singers control their vocal tracts when singing (Bergevin et al., 2020).

Before considering dual-pitch production we need to understand how normal single-pitch singing works. When we sing, the vocal cords in our larynx open and close periodically at a particular frequency (the glottal-pulse rate), and this frequency determines the pitch of the note that we produce. However, we also produce harmonics with frequencies that are multiples of this fundamental frequency. Moreover, the waveform produced by this combination of frequencies is filtered by the resonances in the vocal tract, which we can adjust by moving our lower jaw, tongue, cheeks and lips to change the effective shape of our vocal tract. This filtering causes different frequencies in the sounds we produce to be emphasised, but it does not usually alter pitch: instead, it determines the timbral quality of the sounds in a way that can be associated with meaning. For example, vowel sounds in the English language can be identified, independent of pitch, because each vowel sound has a distinctive pattern of peaks in its frequency spectrum.

In the brain, different frequencies are processed in different neural channels. For a periodic input sound, the fundamental frequency and the first ten or so harmonics are each processed by a different neural channel. However, the neural channels that process higher harmonics handle more than one harmonic, and interactions between these produce oscillations at the same rate as the fundamental frequency. A prominent model put forward in 1994 posits two mechanisms for pitch perception (Shackleton and Carlyon, 1994): at low frequencies, the pitch is conveyed by which neural channels are active, with each channel corresponding to a multiple of the pitch value; at high frequencies, the pitch depends on the temporal pattern produced by interacting harmonics. In general, when we hear a sung note, these 'place' and 'temporal' coding mechanisms reinforce one another and contribute to perception of the same pitch. A sung vowel with a given pitch will contain low harmonics represented in separate frequency channels that represent multiples of the fundamental frequency, and high harmonics that interact in high-frequency channels to produce oscillations at the same rate as the fundamental frequency.

From first principles, there are a number of possible ways of ways of producing a dual pitch. Birds can sing at two different pitches because they have two oscillators in their equivalent of the larynx (Riede and Goller, 2010), but there is no evidence for a similar mechanism in humans. It might also be possible, in principle, for nonlinear oscillation in the larynx to produce a complex signal comprising two distinct pitches, but again there is no evidence for this. In the latest research, Bergevin et al. carried out careful MRI work, which suggests that Tuvan singers use their larynx just like a typical singer, but they also create an extra pitch by controlling the shape of their vocal tract. Specifically, they create a shape that filters out many but not all of the higher harmonics (Figure 1). Bergevin et al. suggest that the range of high frequencies that remains is so narrow that it does not contain enough harmonics to produce oscillations at the fundamental frequency, as usually happens. The result is a new high pitch (determined by the shape of the vocal tract) along with a more typical sung pitch (determined by the lower harmonics).

**Figure 1.**
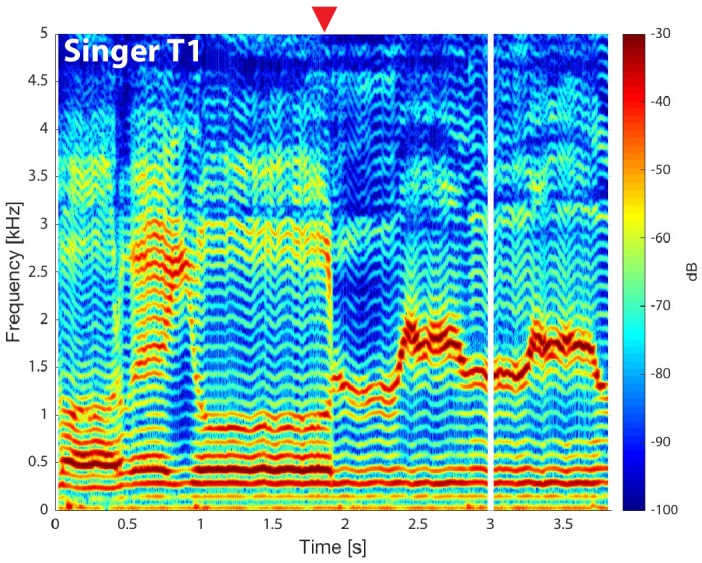
Normal and overtone singing. The output of a Tuvan throat singer as a function of time (horizontal axis) and frequency (vertical axis), as measured by Bergevin et al.; colour is used to represent the intensity of the output, with red being high and blue being low (see colour bar). For the first two seconds we hear a single low pitch (at a frequency of roughly 100 Hz, which is close to the musical note G2): this corresponds to the harmonics that can be seen at multiples of this frequency. After two seconds (to the right of the red arrow), the singer alters the vocal tract to emphasise a narrow band of harmonics between 1 kHz and 2 kHz (shown in red): this emerges as a second higher pitch (which is higher than the musical note B5) that adds a whistle-like sound to the low pitch. It can be seen that the singer is also able to vary the frequency range (and associated pitch) of this band of higher harmonics. Bergevin et al. used MRI to show the changes in the vocal tract that are responsible for the emergence of the frequency band and associated pitch. CREDIT: Bergevin et al., 2020.

The study of Bergevin et al. focuses on a style of singing called khoomei, but this is just one of a number styles practised in Tuva and beyond, so there is plenty more ground to cover for researchers interested in the biomechanics of throat or overtone singing.

## References

[bib1] Bergevin C, Narayan C, Williams J, Mhatre N, Steeves J, Bernstein JGW, Story B (2020). Overtone focusing in biphonic Tuvan throat singing. eLife.

[bib2] Leighton R (1991). Tuva or Bust!: Richard Feynman's Last Journey.

[bib3] Riede T, Goller F (2010). Functional morphology of the sound-generating labia in the syrinx of two songbird species. Journal of Anatomy.

[bib4] Shackleton TM, Carlyon RP (1994). The role of resolved and unresolved harmonics in pitch perception and frequency modulation discrimination. Journal of the Acoustical Society of America.

